# Mechanism of plasma electrolytic oxidation in Mg3ZnCa implants: a study of double-layer formation and properties through nanoindentation

**DOI:** 10.1038/s41598-024-58008-8

**Published:** 2024-03-28

**Authors:** S. Lashkarara, A. fazlali, K. Ghaseminezhad, C. Fleck, M. Salavati

**Affiliations:** 1 Fachgebiet Werkstofftechnik/Chair of Materials Science & Engineering, Institute of Materials Science and Technology, Faculty III Process Sciences, Technische Universität Berlin, Straße des 17. Juni 135, 10623 Berlin, Germany; 2 Chemical Engineering Department, Technical and Engineering Department, Arak University, Sardasht Square, Arak, 38156879 Iran; 3 Electrical Engineering Department, Technical and Engineering Department, Arak University, Sardasht Square, Arak, 38156879 Iran

**Keywords:** Plasma electrolytic oxidation, Double layer, Nanoindentation, Hardness, Elastic module, Engineering, Materials science

## Abstract

Plasma electrolytic oxidation (PEO), applied to light metals such as titanium, aluminum, and magnesium, creates a two-layer coating and has become increasingly important in metal coatings. However, due to the high voltage and temperature of the process, no online instrument could monitor the underlying mechanism. This paper presents a new image proving that the surface of PEO-coated Mg3ZnCa boiled during the process and argues that three hypotheses are involved in the PEO mechanism based on boiling caused by tolerating high voltage during the PEO process, which could explain the current‒voltage diagram of the process. Finally, nanoindentation was used to measure the elastic module and hardness of the PEO layers. The nanoindentation test results revealed the similarity of the elastic module of the outer porous layer and the primary alloy, with values of 40.25 GPa and 41.47 GPa, respectively, confirming that the outer porous layer corresponds to the cold plasma-gas phase formed during the PEO process.

## Introduction

PEO, also referred to as plasma electrolytic polishing (pep) or microarc oxidation (MAO), is a modern method for coating light metals such as Mg, Al, Ti, and Zr^1–4^. This method is based mainly on electrochemical anodizing^5^, while in PEO, the power supply provides more electrical energy to the system. The final surface covered by this method is a ceramic layer, which shows much better properties, such as corrosion resistance, mechanical properties^6^, and homogeneous coverage, than free forms^7^.

In addition to procedural technology, there is increasing focus on the developmental mechanisms of PEO coatings and their microstructure and characteristics. The coatings generated on magnesium alloys comprise a pair of sublayers^8^; the layer closest to the substrate is the ion-releasing and oxide-forming part, and the next layer is the plasma-gas layer. Many different parameters are involved in this process^9^, such as the type and shape of the substrate, electrolyte concentration^10^, temperature, voltage, and process duration^11^. Since the process occurs quickly at high voltages and temperatures, setting online measurements for recording process flow is impossible during PEO, making process analysis more complicated. Thus, most related studies have reported the results of various discrete studies, which are planned to discover the mechanism of this process based on the currently available scientific knowledge^12^.

**Figure 1:**
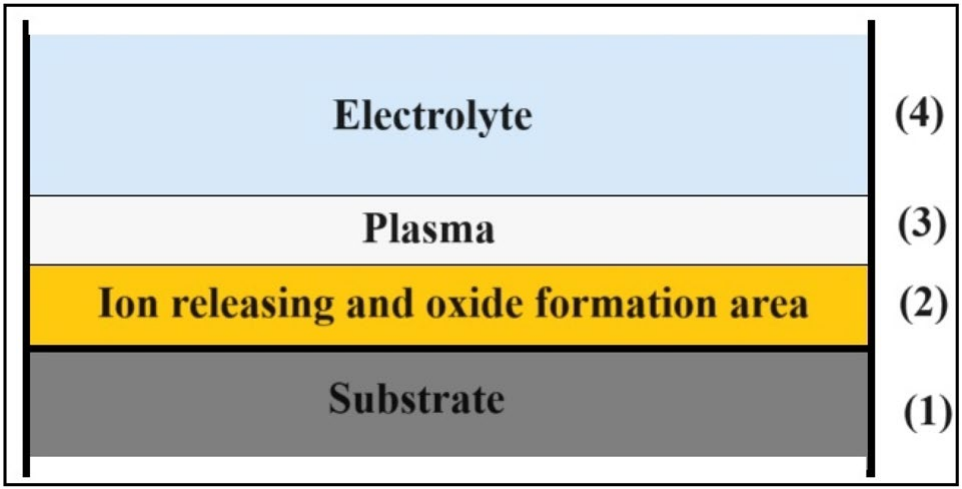
A schematic of the substrate used during the PEO process.

Generally, different researchers have focused their studies on these sections, as described previously Fig. 1.

The PEO process is generally complicated and depends on all the involved parameters, such as electrical power, type of implant, and time. However, the effect of these parameters on the formed coating layer is controversial, and many researchers have published experimental results to clarify all the dependencies in the system. On a smaller scale, a few studies are arguing about the possible mechanism occurring during the PEO process. Figure 1 presents a schematic for the different systems considered for the mechanism hypothesis. Here, the ion-releasing and oxide-forming areas are coded as in section 2, and the plasma film is coded as in section 3. Kellogg^13^ discussed this phenomenon in section 3 and considered that a high voltage leads to gas ionization at the water-steam layer, which initially forms the plasma-gas layer. He assumed that this ionization process causes conductivity in the plasma-gas layer. Similarly, Vana et al.^14^ explained that the ionized water layer partially causes an electric current in the plasma layer. Several other studies have expressed their hypothesis regarding section 2^15^, where they considered section 2 to be a vapor film ionized in an electric field and used it to make the plasma. In this theory, sections 2 and 3 consist of electrolyte and substrate ions, making the PEO process possible.

Electrolyte bridge^16^ is the theory that explains why the plasma-gas layer does not have a constant thickness around the substrate. Moreover, the ponderomotive forces pull the electrolyte toward the substrate. The thickness of the plasma-gas layer decreases when the electrolyte approaches the surface of the substrate. When the electrolyte is close enough, an electrolyte bridge is formed, leading to boiling of the surface, based on Joule heating. Sinkevitch^16^ also thought about explosive boiling in this regard. Moreover, he hypothesized that the plasma-gas layer vibrates during the process.

The Steamer theory describes the process based on the increasing kinetic energy of electrons caused by high voltage^17,18^. Exciting electrons transfer high energy to the next electrons and ionize the molecule. The released electrons create a conductive channel between the electrolyte and the surface of the substrate. At the end of the channel, on the surface of the substrate, an explosion leads to the dispersion of the melted metals.

In other literature, sections 1 and 2 in Fig. 1 were discussed to explain the PEO process, and the authors believe that the process includes surface melting, surface melting-oxidizing, or no melting of the surface. Plotnikov et al.^19^ believe that the PEO mechanism involves melting and oxidizing. As explained, a high substrate temperature results in the formation of bubbles on the surface. A high electric field ionizes the gas inside it and creates high-temperature plasma that melts the oxide layer on the surface of the substrate. The expansion of the bobble results in a shock wave, which will return to the interface between the plasma-gas layer and electrolyte reflectively, which presses the bubble and causes it to collapse. Up to this level, a void replaces the bubble, the ions inside it react with the surface of the substrate, and an oxide layer is formed. Based on this theory, the PEO process occurs when the oxide removal rate is comparable to the rate of oxide formation. Finally, bubbles cover the entire substrate, but the removal rate of the peaks is greater than that of the other parts.

Vana et al.^14^ suggested that glow discharges exist in the plasma layer. They first melt parts with a thinner plasma-gas layer over them, which rapidly smooths the sharp roughness of the substrate. As the surface becomes smoother over time, the removal rate decreases. Considering that PEO is an electrochemical process, researchers argue that no melting occurs during the process^15^. Due to this idea, electrochemical dissolution is the main reason for the PEO mechanism. The thickness of the plasma layer is less than that of the peaks and more than that of the voids. Therefore, the current density is greater than that of the other peaks. Consequently, the removal rate is greater for the peaks, which would soon be smoother.

This paper introduces three novel PEO mechanism hypotheses that discuss boiling of the surface during PEO to elucidate the temperature increase and expansion of the bubble surface observed during the boiling phase. SEM images proved that boiling bobbles were lifted on the Mg3ZnCa PEO-coated implant. The hypotheses introduce the role of component consumption in electrochemical reactions, the thermodynamic effect of the parameters involved in boiling, and the electrical role of the gas bubbles and materials in the reaction. Finally, analyzing the nanoindentation test results showed similar elasticities between the outer layer and the primary alloy, clarifying that the outer porous layer was the cold plasma-gas phase during the PEO process.

## Experiments

### Materials and methods

The main substrate for further PEO study was formed by combining magnesium, zinc, and calcium and casting them as Mg3ZnCa. To prevent oxidation, the melting and casting processes take place under the protection of argon shielding gas in a steel mold. The molten mixture is poured into a cylindrical mold measuring 200 mm in height and 40 mm in diameter when the temperature reaches 750 degrees Celsius. Before being utilized in this study, the chemical composition of the alloy was verified using an inductively coupled plasma (ICP) spectroscopy test. The average chemical composition of the ingots determined via ICP spectroscopy was as follows: magnesium at 95.85 ± 0.6%, zinc at 3.12 ± 0.34%, and calcium at 1.07 ± 0.21%. All the values are reported in weight percentage. The final implant was precisely cut into a rectangular shape measuring 10 × 10 × 5 mm using the wire cut method to facilitate the research.

The steps to create the electrolyte solution for the PEO process and device configuration were as follows: The electrolyte solution was prepared as a mixture of 10 g of Na_3_PO_4_·12H_2_O (molecular weight = 380.13, Merck), 9 g of Na_2_SiO_3_·5H_2_O (molecular weight = 212.14, Sigma–Aldrich), and 1 g of KOH (molecular weight = 56.11, Merck). This mixture was subsequently diluted to a total volume of 1 L. The experimental device was configured for a 1000 Hz frequency, 50% duty cycle, and 450 V for 420 s.

To investigate the surface of the PEO-coated sample, scanning electron microscopy (SEM) was performed using a HIT-S4160 instrument with an electron beam energy of 20 keV. For sample preparation for capturing SEM images of the bursting bubbles, the PEO-coated samples were kept in liquid nitrogen for approximately one hour. Then, the sample was broken by hard-hitting. For the SEM cross-sectional image and nanoindentation test, the PEO-coated sample was fixed in epoxy resin and cut. Then, the surface was polished with 400, 800, 1200, and 4000 sandpapers, and finally, diamond suspensions 3 and 1 µm in length were used to reach a shiny mirror surface.

The nanoindentation tests were performed by a Hysitron TI950 TriboIndenter (Bruker, MA) instrument consisting of a Berkovich diamond tip and a scanning probe microscope (SPM) at room temperature. To explore the hardness and elastic modulus of the layers by nanoindentation, a rectangular area was selected in the cross-section of the PEO-coated specimen such that the tip of the nanoindentator could penetrate deep into 7 rows- 10 columns positions (7 columns to involve substrate, inner layer, outer layer, resin, because the coting thickness of the layers is fluctuating. Also 10 rows were planned for 10 repeats of the 7-column test). Then the machine was run for the sufficient time. When the results became ready, the graphs were draw, and NI marks were checked under the SEM. Those columns that involved NI marks which were placed in pores or borders and could not actually be countable as an accurate measurement or if the pore was suspected to penetration of the resin in it were ignored. Five 7-column sets of tests were remained that depicted accurate sets of measurements. These were used for the discussion part.

SEM was used to study the surface of the specimen before and after nanoindentation. Additionally, ImageJ 1.51 software from the National Institutes of Health, Bethesda, MD, was used to measure the thickness of the layers.

In this method, the hardness and elastic modulus are calculated as follows:

1 $$H=\frac{F}{{A}_{c}}$$

2 $${A}_{c}=24.56{h}_{c}^{2}$$

3 $$E=\frac{1}{2}\sqrt{\frac{\pi }{{A}_{c}}}S$$

in which F is the maximum load. A_c_ is the area of the maximum load, and h_c_ is the depth of the maximum load. E is the elastic modulus, and S is the slope at which unloading starts. The slope of the first 10 percent of the unloading curve is fitted to calculate S.

## Results and discussion

**Figure 2:**
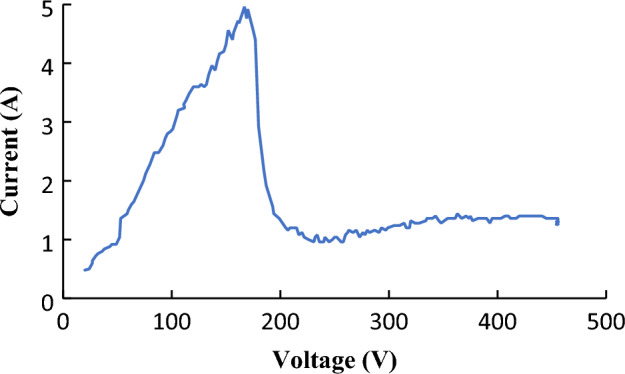
Voltage-current diagram of the PEO process for Mg3ZnCa.

The distinction between the PEO process and anodization lies in the applied voltage range. A high voltage causes the plasma process. During the first 160 s, Mg3ZnCa reached a maximum voltage of 450 V from the starting point at zero volts. The specimen’s surroundings became blurry. By starting the process, a few sparks were replaced by almost stable sparks, which turned into fewer but brighter sparks. Considering the fall of the current coming with the first vision of the sparks being the start moment of the PEO process, it could be concluded that PEO starts almost before halfway through the maximum settled voltage. Alteri et al.^20^ defined the maximum point of the I-V as the breakdown voltage and the point at which the sparks become stable as the discharge or midpoint voltage. Based on Fig. 2, the breakdown voltage is 170 V, established at the 60th second, and the discharge voltage is 202 V.

**Figure 3:**
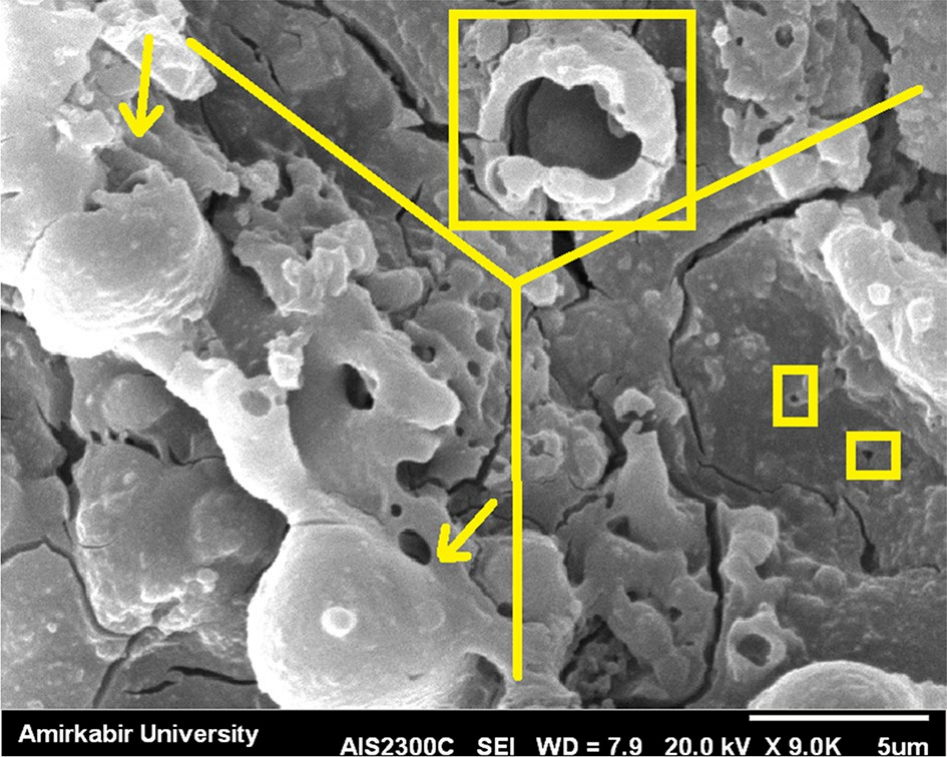
Image of the surface of the broken PEO coating layer.

Figure 3 shows an image of the surface of the coating layer after the PEO process. Several hypotheses^18,21,22^ have explained the possibility of melting, boiling, or volcano eruption of the implant surface during the PEO process. Figure 3 shows a cooled bubble during bursting. As shown in Fig. 3, the outer layer was removed due to the low temperature and the hitting force; however, different layers that remained stepwise above each other were visible. In section a of Fig. 3, a cooled burst bubble proves the boiling theory of the surface. The two spheres in section c could also be caused by boiling. The outer layer was removed in the middle of section b. Previous literature reported that the two tiny holes in this section b could be discharge channels^19^.

This paper presents three hypotheses for bursting boiling bubbles on a sample surface. The first reason involves the interaction and depletion of the implant material’s bubble surface with the electrolyte, which occurs as the bubble surface is utilized, ultimately leading to bubble bursting. Due to the polarity of the high electrical field, the product is absorbed by the implant surface, and layering is performed. The main reactions involving Mg in a PEO process are as follows^23^:

$$Mg\to {Mg}^{2+}+{2e}^{-}$$

$${Mg}^{2+}+2O{H}^{-}\to Mg{(OH)}_{2}\downarrow$$

$$Mg{(OH)}_{2}\to MgO\downarrow +{H}_{2}O$$

$$2Mg+{O}_{2}\to 2MgO\downarrow$$

MgO and Mg(OH)_2_ form due to the presence of water molecules in electrochemical reactions, but the formation of other phases (such as Mg_2_SiO_4_ and MgSiO_3_) depends on the electrolyte ions, which contain silicate, for example^9,22^. The oxidative energy density of Mg in the presence of H_2_O molecules at high temperatures linearly increases from 130 to 360 kJ/mol as the temperature increases^24^. These reactions occur continuously during the PEO process, and new phase formation changes the position of the molecules in the boiled bobble shell, which leads to bursting^25^.

The second reason is that during the boiling of the specimen surface, the boiling bubbles are not necessarily in thermodynamic equilibrium^26^. Considering a stable boiling bubble, there is a balance between the pressure force of the gas inside the bubble and the liquid surrounding it and the tension force of the bubble surface so that the bubble stays stable. An equation for this balance is Eq. (4), written based on the hemisphere cross-section of the bubble. The pressure of the gas inside the bubble *P*_*ν*_ corresponds with the temperature of the gas inside the bubble. Since the working electrode is connected to the power supply, the gas temperature inside the bubble should be greater than the temperature of the electrolyte connected to the cooling system. During conduction through the thickness of the bubble shell, when the gas inside the bubble loses heat and cools, the gas inside the bubble contracts, and as a result, the bubble bursts.

4 $$\pi {r}^{2}\left({P}_{v}-{P}_{l}\right)=2\pi r\sigma$$

Finally, Ohm-Low explains the third hypothesis. The Ohm law is presented in Eq. (5). In this equation, the impedance is represented by a complex number with real and imaginary parts of R and jX, which are expressed in Eqs. (6 and 7), respectively, based on their involved parameters. Every part of a circuit has a specific impedance, the real part of which is the resistance (R), which corresponds to the material type and distance and the reverse of the area represented by ρ, l, and A_R_^−1^ in Eq. (6). Based on many experiments, Alteri et al.^20^ concluded that the formation of a vapor film around a specimen as the working electrode is mandatory for the PEO process. When a bubble is formed on the implant's surface, this distance decreases as long as the bubble's diameter. This means that the thickness of the vapor film fluctuates. The surface also expands while a bubble grows. As a result, the resistance decreases, increasing the current and temperature. An increase in the surface temperature of the bubble shell causes the bubble to expand and burst. This vapor film could also act as a capacitor, which could be discharged in response to changes in various parameters, such as the material type, quantity, and thickness of the film, and the sparks became obvious. Equation (8) describes the effective parameters of a capacitance.

5 $$V=(R+jX)I$$

6 $$R=\frac{\rho l}{{A}_{R}}$$

7 $$jX=j2\pi fL+\frac{1}{j2\pi fC}$$

8 $$C={\varepsilon }_{r}{\varepsilon }_{^\circ }\frac{A}{d}$$

9 $$\frac{1}{{C}_{total}}=\sum \frac{1}{{C}_{i}}$$

For a circuit containing electrolytes, the first part of Eq. (7), including the inductance, could be negligible compared to the second part containing capacitance (C). All the gas bubbles could be considered capacitances during the process; oxygen and hydrogen bubbles would form in the electrolyte and take place around the anode and cathode, respectively^27^. They would pass through each other, and in some moments, they could be next to each other in the discussed film. Thus, the circuit would have a series capacitance. This attitude could be extended by the addition of other material vapors, such as H_2_O. On the other hand, the anode is coated with a thin SiO_2_ film^28^, which provides a new capacitance. The thickness of the SiO_2_ film and the PEO coating layer increase during the process. Consequently, the total amount of these capacitances calculated by Eq. (9) fluctuates. This could explain many of the small fluctuations in the graph in Fig. 2 after 200 V. This theory also explains the uneven structure of the surface coated by the PEO method.

**Figure 4:**
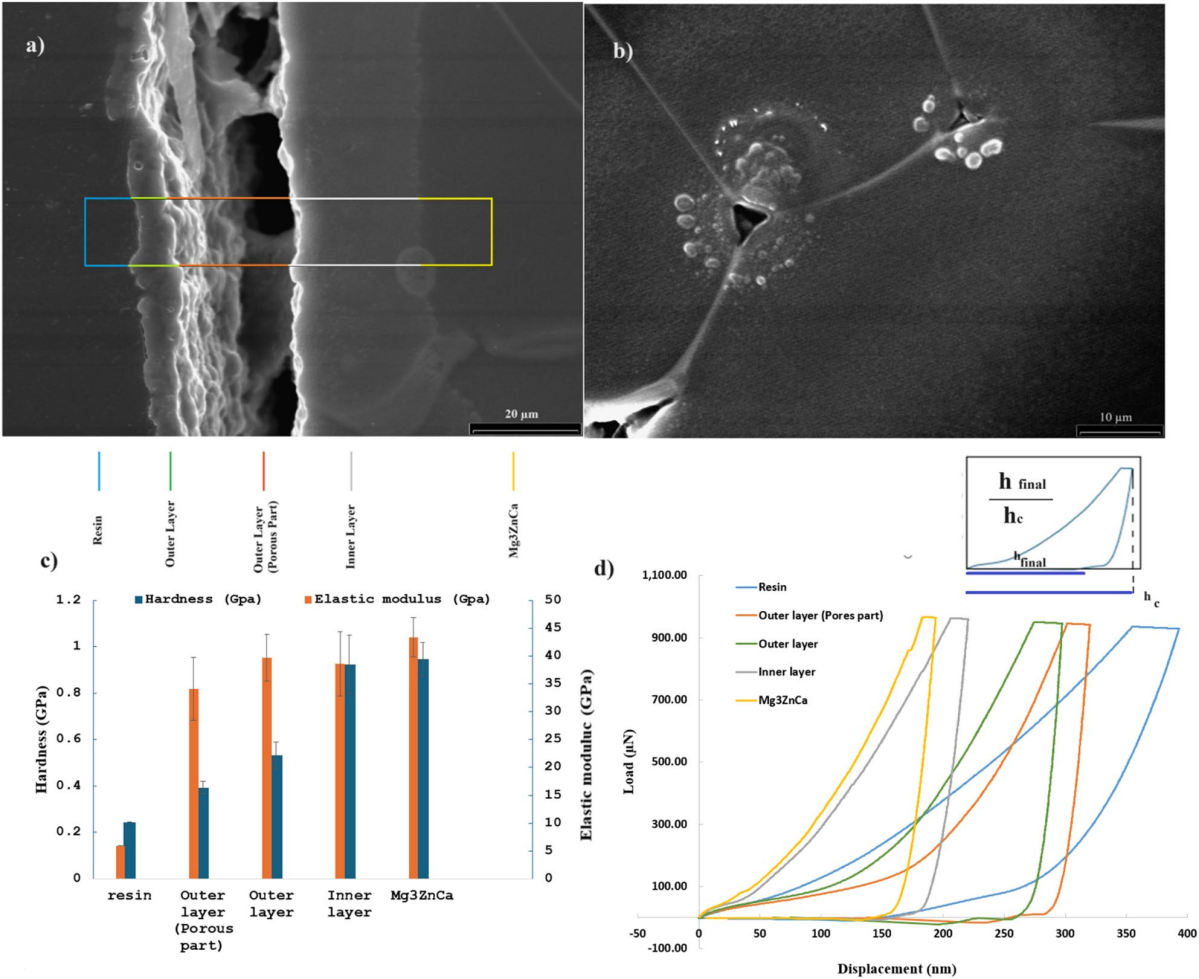
Analysis of the different layers of the Mg3ZnCu implant coated with PEO material. (**a**) SEM image of the cross section. (**b**) Nanoindentation test tips under the microscope. (**c**) Hardness–elastic modulus diagram of the different layers. (**d**) Load–displacement diagram.

Figure 4a displays a cross section of the layer coated by the PEO method, as observed via SEM. The PEO coating layer on the implant surface was divided into two main parts. The surface adjacent to the implant is a dense layer without pores, which effectively protects against corrosion and creates hardness. This layer forms the base of the bubbles during boiling. The next layer is a porous layer connected to the dense layer. The porous layer could be a cooled gas plasma layer over the boiling bubbles in contact with the electrolyte. This figure shows how these layers are connected by connections such as bridge columns, which are visible deep in the porous region between the layers. The underneath vision of the outer layer also presented a rough surface. These could also be from bubbles on the boiling surface during the PEO process. The thickness of the inner layer in this figure is about 20 µm, which looked to be almost not changing along the edge. The outer porous layer thickness is fluctuating, but it is thicker than the inner layer. The outer layer was at least about 7 µm. These numbers are not the same along the edge while the edge is fluctuating due to electrochemical reactions and boiling. The rectangular area was selected to examine the hardness and elastic modulus of each sample layer, including the resin, outer layer, outer porous layer, inner layer, and finally, the main implant parts. Different colors correspond to different layers, as it can be seen and discuss in Fig. 4c.

Figure 4b shows triangles as empty pyramids whose base remained adjacent to the nanoindentation test tip in substrate. There are some prominent circular spaces around the empty pyramid spaces. This occurs because the nanoindentation test tip deepens the surface. When the tip moves inside the surface, the molecules are placed under it, and the adjacent molecules are pressed. Since there is not much space between the solid molecules of the Mg3ZnCa implant, a crescent around the test site can form. There are also some tiny bubbles near each test mark. These differences could be caused by the back-up movement of the test tip through the surface. To clarify, after the tip deepens enough and starts to come back, almost no space remains between the tip and the solid molecules. Therefore, there would be a vacuum between the molecules that tolerate the pressure of the guest molecules removed by the tip. All these molecules are evacuated until the tip separates from the surface. The molecules with strong intermolecular bonding^29^ would also be released and returned as much as possible. However, their new place was not their original place before the test, not their maximum upward place during this process. In small local spaces, connected molecules make several tiny bubbles adjacent to each other. Therefore, they would be visible as tiny bubbles around the mark. The edges of the nanoindentation mark are not as straight as expected based on the shape of the machine tip, but rather, the shape is curved, which shows that molecules on the surface of the empty pyramid tend to take back their original place and use the hollow space remaining on the tip. Moreover, different nanoindent place marks are connected to the next mark with a line. These cracks are known as fracture toughness. Fraction deformation could involve very complex analyses and measurements^30^. In this case, the deformation of the crystalline structure by each nanoindent footprint results in a similar near-one deformed crystalline structure, and the cracks become connected. This crystalline structure deformation depends on the elastic and plastic behavior of the implant, as well as the indenter geometry^31^.

The hardness and elastic modulus of the different layers were calculated using the Oliver–Pharr method and are displayed in Fig. 4c. The elastic modulus of the outer layer (without pores) is similar to the elastic modulus of the implant, which supports the idea that the second layer of the coating is formed by cooling the plasma and gas around the implant after the PEO process. Since the plasma zone could be almost as dense as the primary alloy^32^, based on boiling theory, as explained earlier, it was predictable that a cooled dense area (the outer layer) could show this high elastic modulus close to the main alloy amount. Thus, this could support the theory that the surface of the initial Mg3ZnCa alloy was melted and boiled, and a plasma-gas layer was generated during the PEO process.

Figure 4d shows the load‒displacement diagram obtained from the analysis of the results of this experiment**.** All the samples exhibited elastic‒plastic behavior, and the softer layers exhibited greater displacements. Kværndrup et al.^33^ explained that if the ratio of h_final_ (the place where the curve cuts the displacement axis) to h_c_ (the maximum displacement that the curve establishes) is more than 0.7, it is proven that piling up happened during the test. Otherwise, the indent mark reports a sink-in. A small schematic diagram in Fig. 4d showed the $\frac{{h}_{final}}{{h}_{c}}$ ratio. According to their studies, piling occurs due to material properties, while sinking commonly occurs in high-concentration samples. This ratio is 0.73, 0.75, 0.86, and 0.89 for the substrate, inner layer, outer layer, and outer porous layer respectively, which suggest piling up result for these layers. This is also exhibited in Fig. 4b, which shows a nanoindentation mark remained in the substrate. Only the curve corresponding to the resin has this ratio equal to 0.37 that is less than 0.7, which is logical since the epoxy was dried and had a higher concentration than the liquid state, which was used for sample preparation. In addition, as could be predicted, the alloy proved to be the hardest, and the resin was the softest. The inner layer, which was a dense layer next to the alloy, had a similar hardness to that of the main implant, but the outer layer with pores was softer than the outer layer without pores and other layers, which was obvious because of the existence of the pores. The lower hardness of the outer porous layer in comparison to that of the main alloy was also reported in previous research^34^.

## Conclusion

Online monitoring of the mechanism during the PEO process is impossible due to the high voltage and temperature. This paper studied the use of a PEO coating on a Mg3ZnCa implant. A gas film forms over the specimen to cause the PEO process to occur. The gas film includes different gas types, such as hydrogen, oxygen, and water, which act as series capacitors. It has been established that surface boiling occurs during the PEO process, forming bubbles that effectively reduce the amount of gas film next to the specimen. A reduction in distance subsequently changes the total amount of capacitance. Discharging of these capacitances could cause sparks. The nanoindentation test proved that the outer layer shares elastic modulus similar to that of the main metal, whereas the inner layer has a similar hardness to that of the substrate. Therefore, this research suggested that the mechanism of the PEO process involves the surface melting, boiling, and subsequent formation of plasma gas. The dense layer is the melt connected to the implant and reacts during the process; vapor causes the porous layer to form, and the plasma phase is created by high heat and participates in the reactions. Nanoindentation remaining under SEM presented circular places around the marks, which happened by moving the places of the molecules to tolerate the force of the nanoindentation test tip. The large circle was caused by molecules that could not access the space for the nanoindentation tip when it was deep into the surface, and the tiny bubbles and curved shapes of the surfaces of the pyramids remained marked by the vacuum formed by raising the tip back up. The remaining nanoindentation marks are connected with a line, which could cause a change in the crystalline structure of the tested surface.

## Abbreviations

F: Maximum load (N)
A_c_: Area of the maximum load (m^2^)
h_c_: Depth of the maximum load (m)
h_final_: Final depth of the indent in load–displacement diagram
E: Elastic modulus (Pa)
S: Slope of the place that unloading is started
$$\pi$$: 3.14
r: Radios of the boiled bubble (m)
${P}_{v}$: Vapor pressure inside bubble (Pa)
${P}_{l}$: Liquid pressure (Pa)
$\sigma$: Surface tension of vapor–liquid interface (Pa)
V: Voltage (V)
R: Resistance (Ω)
I: Current (A)
$$\rho$$: Resistivity (Ω⋅m)
l: Length of resistance (m)
${A}_{R}$: Cross-sectional area of resistance (m^2^)
X: Reactance (Ω)
j: Imaginary unit
f: Frequency (Hz)
L: Inductance (H)
C: Capacitance (F)
$${\varepsilon }_{r}$$: Relativity permittivity (F/m)
$${\varepsilon }_{^\circ }$$: Vacuum permittivity (F/m)
A: Capacitance plate area (m^2^)
d: Distance between plates (m)
${C}_{total}$: Total equivalent capacitance (F)
${C}_{i}$: Capacitance made from one type of material (F)
